# Activation of Ca^2+^‐activated Cl^−^ channel ANO1 by localized Ca^2+^ signals

**DOI:** 10.1113/jphysiol.2014.275107

**Published:** 2014-12-15

**Authors:** Xin Jin, Sihab Shah, Xiaona Du, Hailin Zhang, Nikita Gamper

**Affiliations:** ^1^ School of Biomedical Sciences, Faculty of Biological SciencesUniversity of LeedsLeedsUK; ^2^ Department of PharmacologyHebei Medical UniversityShijiazhuangChina

## Abstract

Ca^2+^‐activated chloride channels (CaCCs) regulate numerous physiological processes including epithelial transport, smooth muscle contraction and sensory processing. Anoctamin‐1 (ANO1, TMEM16A) is a principal CaCC subunit in many cell types, yet our understanding of the mechanisms of ANO1 activation and regulation are only beginning to emerge. Ca^2+^ sensitivity of ANO1 is rather low and at negative membrane potentials the channel requires several micromoles of intracellular Ca^2+^ for activation. However, global Ca^2+^ levels in cells rarely reach such levels and, therefore, there must be mechanisms that focus intracellular Ca^2+^ transients towards the ANO1 channels. Recent findings indeed indicate that ANO1 channels often co‐localize with sources of intracellular Ca^2+^ signals. Interestingly, it appears that in many cell types ANO1 is particularly tightly coupled to the Ca^2+^ release sites of the intracellular Ca^2+^ stores. Such preferential coupling may represent a general mechanism of ANO1 activation in native tissues.

**Abbreviations**
ANO: anoctamin
AP: action potential
BAPTA: 1,2‐bis(2‐aminophenoxy)ethane‐*N,N,N′,N′*‐tetraacetic acid
CaCC: Ca^2+^‐activated Cl^−^ current
CRAC: Ca^2+^ release‐activated channel
DIDS: 4,4′‐diisothio‐cyanostilbene‐2,2′‐disulfonic acid
DRG: dorsal root ganglion
EGTA: ethylene glycol‐bis(2‐aminoethylether)‐*N*,*N*,*N′*,*N′*‐tetraacetic acid
ER: endoplasmic reticulum
GPCR: G protein‐coupled receptor
HEK293: human embryonic kidney 293 cell line
ICC: interstitial cells of Cajal
IP_3_R: inositol 1,4,5‐trisphosphate receptor
βMCD: methyl‐β‐cyclodextrin
NFA: niflumic acid
NMDA: *N*‐methyl‐d‐aspartic acid
NPPB: 5‐nitro‐2‐(3‐phenylpropylamino) benzoic acid
PAR‐2: protease‐activated receptor 2
PLA: proximity ligation assay
PLC: phospholipase C
P2Y: purinergic G protein‐coupled receptor
RyR: ryanodine receptor
SOCE: store‐operated Ca^2+^ entry
STIC: spontaneous transient inward currents
STOC: spontaneous transient outward currents
TMEM16: transmembrane protein 16
TRPV1: transient receptor potential cation channel subfamily V member 1
VGCC: voltage‐gated Ca^2+^ channel

## Introduction

Ca^2+^‐activated Cl^−^ channels (CaCCs) play crucial roles in numerous physiological processes including epithelial transport, smooth muscle contraction and sensory processing. In epithelia CaCCs are important for Ca^2+^‐activated Cl^−^ secretion and mucus production (Danahay *et al*. 2002; Galietta *et al*. 2002; Scudieri *et al*. 2012). In smooth muscle CaCCs mediate agonist‐induced contractions, e.g. in response to noradrenaline (norepinephrine), endothelin or histamine (Klockner & Isenberg, 1991; Van Renterghem & Lazdunski, 1993; Vennekens *et al*. 1999). Smooth muscle cells accumulate high intracellular Cl^−^ concentrations due to Cl^−^–HCO_3_^−^ exchange and Na^+^–K^+^–Cl^−^ cotransport (Aickin & Vermue, 1983; Owen, 1984; Meyer *et al*. 2002) providing the necessary driving force for depolarizing Cl^−^ currents. Activation of Cl^−^ channels in these cells is therefore excitatory and stimulates contraction. In the nervous system functional CaCCs are best characterized in neurons with various sensory functions, such as olfactory neurons (Kleene & Gesteland, 1991; Lowe & Gold, 1993), photosensitive rods and cones (Bader *et al*. 1982; Maricq & Korenbrot, 1988; Barnes & Hille, 1989), taste cells (Taylor & Roper, 1994) and somatosensory neurons (Liu *et al*. 2010; Cho *et al*. 2012; Jin *et al*. 2013). It is thought that in these neurons CaCCs amplify signals generated by Ca^2+^‐permeable channels in response to the respective sensory events. Rises in intracellular Ca^2+^ concentration ([Ca^2+^]_i_) activate CaCCs and produce depolarization as most of these neurons accumulate high intracellular Cl^−^ concentrations (Liu *et al*. 2010; Cho *et al*. 2012); in this regard sensory neurons are similar to smooth muscle cells but are different from most CNS neurons which in adult mammals have very low concentrations of intracellular Cl^−^ (Delpire & Staley, 2014). In addition to the amplification of sensory signals, CaCCs may contribute primary depolarizing currents in response to some of these signals (e.g. in response to inflammatory mediators or heat in pain‐sensing or ‘nociceptive’ neurons (Liu *et al*. 2010; Cho *et al*. 2012; Jin *et al*. 2013; Lee *et al*. 2014)).

The molecular nature of CaCCs has been elusive for many years; while some members of CLC and bestrophin families were originally considered as candidates, no consensus regarding their involvement emerged (Ferrera *et al*. 2010). More recently a new family of anion channels, the anoctamins (ANO or TMEM16), was suggested as a likely candidate with ANO1 (TMEM16A) (Caputo *et al*. 2008; Schroeder *et al*. 2008; Yang *et al*. 2008) and ANO2 (TMEM16B) (Stephan *et al*. 2009; Stohr *et al*. 2009) subunits identified as bona fide CaCCs (see Pedemonte & Galietta, 2014 for review). For example, ANO1 (TMEM16A), reconstituted in an expression system, reproduced key features of native CaCC currents such as higher permeability to I^−^ over Cl^−^, micromolar Ca^2+^ sensitivity, outwardly rectifying voltage dependence and sensitivity to the CaCC blockers 5‐nitro‐2‐(3‐phenylpropylamino)benzoic acid (NPPB), niflumic acid (NFA) and 4,4′‐diisothio‐cyanostilbene‐2,2′‐disulfonic acid (DIDS) (Hartzell *et al*. 2005; Schroeder *et al*. 2008; Yang *et al*. 2008; Liu, 2014). Accordingly, ANO1 was found to mediate CaCC currents in epithelia and smooth muscles (Huang *et al*. 2009; Rock *et al*. 2009). In addition, ANO2 has been identified as a CaCC subunit in the cilia of olfactory sensory neurons (Stephan *et al*. 2009; Billig *et al*. 2011). It has to be pointed out that some native CaCC currents may be mediated by other channels not related to ANO family (e.g. by Best1; see below).

Anoctamins share no significant sequence similarity with any other ion channels or other membrane proteins. A topological model with at least eight transmembrane (TM) domains, cytosolic N‐ and C‐termini and a re‐entrant loop between TM5 and TM6 (probably pore‐lining) has been proposed (Yang *et al*. 2008; Ferrera *et al*. 2010) but structure–function relationship data for anoctamins are still sparse. Similarly little is known about the regulatory or other proteins that might interact with anoctamins and regulate their function. Even the activation mechanism(s) of ANO‐mediated CaCCs are understood only in very general terms. Moreover, what becomes known is often controversial; for example, calmodulin was suggested to bind to ANO1 and modulate its activation by Ca^2+^ (Tian *et al*. 2011; Vocke *et al*. 2013) or anion permeability (Jung *et al*. 2013) but these findings are being disputed (Terashima *et al*. 2013; Yu *et al*. 2014). Yet the understanding of the mechanisms of activation and molecular interactions of ANO channels is paramount for unravelling their physiological roles.

**Figure 1:**
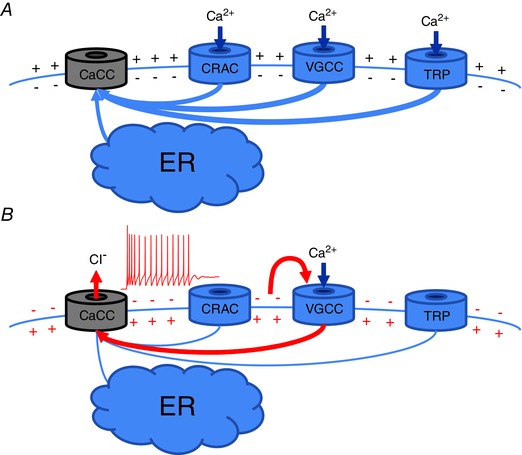
**Simplified schematic diagram illustrating possible relationships between Ca^2+^‐activated Cl^−^ channels (CaCCs) and sources of intracellular Ca^2+^ in cells** *A* depicts CaCC activation by Ca^2+^ release from the endoplasmic reticulum (ER) and Ca^2+^ influx through the Ca^2+^ release‐activated channels (CRACs), voltage‐gated Ca^2+^‐channels (VGCCs) and non‐selective cation channels (labelled ‘TRP’ here but theoretically can be any other non‐selective cation channel). *B* depicts a possible mechanism for the positive feedback loop in the case of close coupling between CaCCs and VGCCs in excitable cells.

ANO1/ANO2 channels and native CaCCs are activated by rises in intracellular Ca^2+^, however, intracellular Ca^2+^ signals in cells can arise from very diverse sources and in response to very different stimuli. Thus, events such as (i) opening of sensory cation channels (such as TRP) or any ligand‐gated cation channels, (ii) activation of voltage‐gated Ca^2+^ channels (VGCCs), (iii) release of Ca^2+^ from intracellular stores, or (iv) store‐operated Ca^2+^ entry (SOCE) all result in intracellular Ca^2+^ transients (Fig. 1 *A*). The question arises: would all these diverse processes uniformly activate CaCCs in a given cell type? Let us consider a pain‐sensing (nociceptive) sensory neuron as an example. Figure 1 outlines in a very simplistic form the possible relationships between the [Ca^2+^]_i_, CaCC activation, membrane potential and activity of an excitable cell. Nociceptive sensory neurons are normally silent and respond only to strong adverse stimuli that can result in tissue damage. Action potentials (APs) that are triggered by such stimuli in the peripheral terminals of nociceptors (or elsewhere along their very long axons) travel to the spinal cord where these neurons synapse. Second order neurons in the spinal cord then relay these peripheral nociceptive signals to the higher brain centres where these signals can be perceived as a sensation of pain. Since CaCC activation depolarizes and excites nociceptive neurons, an action that may result in pain sensation (Liu *et al*. 2010; Cho *et al*. 2012; Lee *et al*. 2014), there must be mechanisms that tightly control coupling of Ca^2+^ sources to CaCC activation in nociceptors to prevent unnecessary AP firing. A need for such mechanism(s) is especially obvious in the case of possible relationships between CaCCs and VGCCs (Fig. 1 *B*). Indeed, if Ca^2+^ influx through the VGCCs (opened, for example, during the AP firing) did reach CaCCs and activate them, the depolarization produced by such activation might in turn further activate VGCCs (Hartzell *et al*. 2005). Arguably, such a scenario must be avoided in nociceptive neurons as such positive feedback loop could result in overexcitable neurons and a chronic pain condition. Similar considerations may apply to other excitable and non‐excitable cells as these also need to maintain fidelity and specificity of their Ca^2+^ signalling. In accord with these conventional arguments, growing evidence suggests that anoctamin‐mediated CaCCs are indeed rather fussy about where the Ca^2+^ is coming from and, as will be discussed below, native CaCCs in various tissues and cell types often display peculiar preference for the particular sources of Ca^2+^.

## Coupling between ANO1 and endoplasmic reticulum; is this a general principle?

There is a growing body of evidence indicating that in multiple cell types CaCCs mediated by ANO1 are particularly well coupled to the Ca^2+^ release from intracellular stores. In early experiments on endogenous CaCCs in *Xenopus* oocytes Criss Hartzell's group electrophysiologically separated what appeared as two distinct types of CaCC currents with different preferences for the Ca^2+^ source. One of these currents was outwardly rectifying, displayed slow, time‐dependent activation and was specifically activated by the IP_3_‐induced Ca^2+^ release from the endoplasmic reticulum (ER) Ca^2+^ stores. Another current had linear current–voltage relationships, lacked slow activation kinetics and was activated by Ca^2+^ influx through SOCE (Hartzell, 1996; Kuruma & Hartzell, 1999). It subsequently turned out that both currents are mediated by the same channel (Kuruma & Hartzell, 2000), later identified as ANO1 (Schroeder *et al*. 2008), which displays different properties depending on the concentration of intracellular Ca^2+^ it is being exposed to. At sub‐micromolar [Ca^2+^]_i_ ANO1 exhibits outward rectification and slow, time‐dependent activation at depolarizing voltages while at [Ca^2+^]_i_ above several micromolar both these features largely disappear (Xiao *et al*. 2011). A recent study added an unexpected twist to this ‘tale of two currents’ suggesting that SOCE activates ANO1 in oocytes not directly but also in an ER‐dependent way (Courjaret & Machaca, 2014). Using a combination of electrophysiology and imaging techniques, the authors suggested that Ca^2+^ entering from the extracellular media through the STIM1–Orai1‐mediated SOCE first needs to be channelled into the ER and then out through the IP_3_ receptors (IP_3_Rs) in order to be able to activate endogenous ANO1 in oocytes. It was further observed that a direct Ca^2+^ influx through STIM1–Orai1 complexes into the cytosol cannot activate ANO1, presumably due to the lack of close proximity between the ANO1 and the STIM1–Orai1 complexes. Thus, it appears that both types of oocyte CaCCs are indeed activated by the Ca^2+^ released from the ER via the IP_3_Rs, but when SOCE is engaged as well, the Ca^2+^ release is stronger, rendering ANO1‐mediated CaCCs less voltage dependent. Although this was not the first study showing preferential coupling of ANO1 channels to the IP_3_R‐mediated Ca^2+^ release (see below), it did complement earlier studies on native CaCCs in oocytes to point to such preferential coupling.

In a separate line of enquiry, Karl Kunzelmann's group suggested that compartmentalization of Ca^2+^ signals is important for ANO1 activation. Specifically, they proposed that functional proximity of ANO1 to the ER‐localized IP_3_Rs is important for the activation of ANO1 channels by the G_q/11_‐ and PLC‐coupled purinergic P2Y receptors in HEK293 cells and oocytes (Barro‐Soria *et al*. 2010; Kunzelmann *et al*. 2012). Such coupling could explain an apparent paradox experimentally observed in HEK293 cells: overexpression of P2Y receptors resulted in an increase in ATP‐induced CaCC current but produced no net increase in the global cytosolic ATP‐induced Ca^2+^ release (since endogenous P2Y receptors are sufficient to produce maximal release). This observation could be explained by an assumption that the increase in P2Y receptor density results in stronger IP_3_ release upon ATP application. This, in turn, engages additional IP_3_Rs in the response. In such a scenario Ca^2+^ imaging with cytosolic Ca^2+^ indicators may not reveal any difference between the P2Y‐receptor‐overexpressing and control cells; yet additional IP_3_R Ca^2+^ release sites could activate more co‐localized ANO1 channels. An additional suggestion for close association between Ca^2+^ release sites and ANO1–CaCCs came from oocyte recordings: in oocytes overexpressing ANO1 and P2Y_2_ receptors, whole‐cell currents activated by the Ca^2+^ ionophore ionomycin displayed an outward rectification, while ATP‐activated currents had linear current–voltage relationships, suggesting higher local Ca^2+^ concentrations near ANO1 channels in the latter case (Kunzelmann *et al*. 2011, 2012).

**Figure 2:**
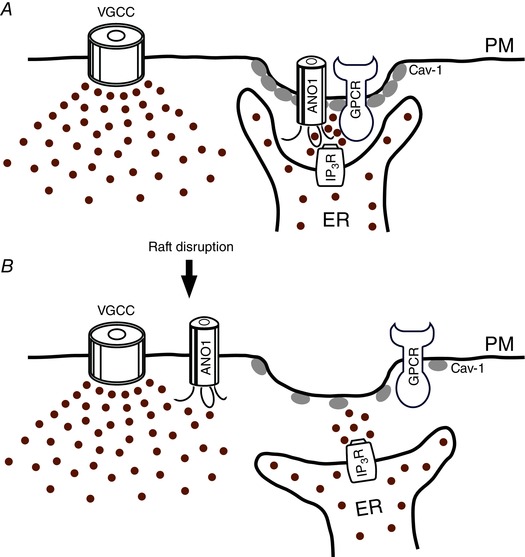
**Simplified schematic diagram of the proposed juxtamembrane arrangements within an ANO1‐containing signalling microdomain in a nociceptive sensory neuron** ANO1, anoctamin‐1; Cav‐1, caveolin‐1; ER, endoplasmic reticulum; GPCR, G protein‐coupled receptor; IP_3_R, inositol 1,4,5 trisphosphate receptor; PM, plasma membrane; VGCC, voltage‐gated Ca^2+^ channels; brown circles represent Ca^2+^ ions. Panel *A* represents control conditions and panel *B* depicts proposed rearrangements after the lipid raft disruption.

In our laboratory we investigated a phenomenon closely related to the one described above; we found a preferential coupling of ANO1 channels to IP_3_Rs in nociceptive sensory neurons. Sensory neurons display robust CaCC currents (Mayer, 1985; Ward & Kenyon, 2000; Andre *et al*. 2003), which are most likely mediated by ANO1 channels (Liu *et al*. 2010; Cho *et al*. 2012; Lee *et al*. 2014). Due to the relatively high intracellular Cl^−^ concentration in the peripheral sensory neurons (*E*_Cl_ in the range of –35 to –40 mV (Liu *et al*. 2010)), activation of CaCCs in these cells produces depolarization and may cause AP firing and, ultimately, pain (Liu *et al*. 2010; Cho *et al*. 2012; Lee *et al*. 2014). Interestingly, it appears that in nociceptors CaCCs are coupled more closely to the IP_3_‐mediated Ca^2+^ release from the ER than to the Ca^2+^ influx via the VGCCs. In earlier studies it was found that about 50% of all dorsal root ganglion (DRG) neurons express VGCC‐coupled CaCCs (Mayer, 1985). However, subdivision of neurons into large (mostly mechano‐sensitive), medium and small (mostly nociceptive) neurons later revealed that while medium and large neurons express VGCC‐coupled CaCCs (Andre *et al*. 2003; Boudes *et al*. 2009; Boudes & Scamps, 2012), in small neurons such coupling is rarely seen (Andre *et al*. 2003; Liu *et al*. 2010; Boudes & Scamps, 2012). At the same time ligands of G_q/11_‐ and PLC‐coupled G protein coupled receptors (GPCRs) such as bradykinin receptor 2 (B_2_) or protease‐activated receptor 2 (PAR‐2) activate CaCCs reliably (Liu *et al*. 2010; Jin *et al*. 2013). Thus, in patch‐clamp recordings from cultured capsaicin‐sensitive DRG neurons we found that activation of VGCCs with voltage pulses rarely resulted in a CaCC current activation, even in same neurons in which B_2_ or PAR‐2 receptor triggering did activate CaCCs (Jin *et al*. 2013). We hypothesized that preferential coupling of ANO1 to the Ca^2+^ released from the ER, rather than to Ca^2+^ influx through VGCCs, may arise from the close juxtaposition of endogenous ANO1 channels with the ER Ca^2+^ release sites and the lack of such proximity between ANO1 and the VGCCs. This, in combination with the low Ca^2+^ sensitivity of ANO1 channels (EC_50_ at negative voltages is ∼2–5 μm; Yang *et al*. 2008; Xiao *et al*. 2011), would make them insensitive to ‘distal’ Ca^2+^ elevations. Differential sensitivity to ‘local’ and ‘global’ Ca^2+^ signals has been successfully probed for Ca^2+^‐activated K^+^ (and some other) channels (Berkefeld *et al*. 2006) with two Ca^2+^ buffers, EGTA and BAPTA. EGTA is a ‘slow’ Ca^2+^ buffer which cannot block fast local Ca^2+^ elevations while BAPTA is a ‘fast’ buffer that is able to block both local and global Ca^2+^ signals (Augustine *et al*. 2003). The two buffers have comparable Ca^2+^ affinities. In DRG neurons intracellular dialysis with 10 mm EGTA did not prevent GPCR‐induced CaCC currents; in contrast, 10 mm BAPTA abolished such currents almost completely. These results suggested close proximity of ANO1 with ER Ca^2+^ release sites. Further supporting this hypothesis, we found that ANO1 could be immunoprecipitated from rat whole DRG lysates using an IP_3_R1 antibody and, reciprocally, IP_3_R1 could be precipitated by an ANO1 antibody. Moreover, we were able to detect close association of IP_3_R and ANO1 channels using a proximity ligation assay (PLA), a highly specific proteomics method that enables optical detection of closely associated proteins (must be within less than 40 nm proximity in order to be detected; Jarvius *et al*. 2006; Soderberg *et al*. 2006). In addition to the above evidence, other reports suggested that (i) GPCRs such as B_2_ receptors may also co‐localize with IP_3_ receptors in junctional plasma membrane–ER microdomains (Delmas & Brown, 2002; Delmas *et al*. 2002; Zhang *et al*. 2013 *b*) and (ii) B_2_ receptors localize to plasma membrane lipid rafts in sympathetic and sensory neurons (Jeske *et al*. 2006; Jeske, 2012; Zhang *et al*. 2013 *b*). In accord with these reports we found that B_2_ and PAR‐2 receptors co‐immunoprecipitated with the IP_3_R and with the lipid raft marker caveolin‐1 (Jin *et al*. 2013). Thus, we hypothesized that a functional signalling unit that produces CaCC‐mediated depolarization in response to inflammatory mediators in nociceptive sensory neurons may be assembled at the plasma membrane–ER junctions. Such junctions are abundant in eukaryotic cells and are increasingly recognized as intracellular signalling ‘hubs’ (Stefan *et al*. 2013). We further hypothesized that plasmalemmal components of the signalling complex (i.e. GPCRs, ANO1) reside within lipid rafts (Fig. 2 *A*). In accord with this idea, membrane fractionation experiments revealed that in DRG neurons ANO1 localized to the same membrane fractions as caveolin‐1, B_2_ receptors and PAR‐2. However, this distribution was disrupted by cholesterol depletion with methyl‐β‐cyclodextrin (βMCD), a procedure known to disrupt lipid rafts (Maekawa *et al*. 1999). Similarly, co‐immunoprecipitation and PLA experiments revealed that the cholesterol depletion disrupted the interaction between ANO1 and IP_3_R1 in DRG neurons. Glutathione S‐transferase (GST) pull‐down experiments using GST‐fused cytosolic domains of ANO1 revealed that both the C‐terminus and the cytoplasmic TM2/3 loop but not the N‐terminus of ANO1 were able to precipitate endogenous IP_3_R1 in DRG preparation. We reasoned that if lipid raft disruption distorts ANO1 membrane localization and interrupts the interaction with IP_3_R1, this may also affect the ability of ANO1 to differentiate sources of intracellular Ca^2+^. Indeed, after the βMCD treatment, activation of neither PAR‐2 nor B_2_ receptor was able to activate CaCC currents in DRG neurones. In contrast, in 50% of such βMCD‐treated neurones we observed CaCC activation coupled to the VGCC activation. Thus, disassociation of the ANO1 signalling complex in a small DRG neuron resulted in loss of functional coupling between the GPCRs and the ANO1 but instead some coupling between ANO1 and VGCCs appeared. We hypothesize that the latter effect is due to the proximity between ANO1 and VGCCs acquired by chance after the raft disruption as both proteins reside in the plasma membrane (in contrast to the IP_3_Rs; Fig. 2 *B*). Consistent with a VGCC–CaCC–VGCC positive feedback loop idea (see Fig. 1 *B*), current clump experiments revealed that most small‐diameter DRG neurons fired single APs in response to strong depolarizing current injections in control conditions; however, lipid raft disruption resulted in overexcitable neurons that fired APs trains. Thus, we proposed a model outlined in Fig. 2: under resting conditions ANO1 in nociceptive sensory neurons resides within signalling complexes at the plasma membrane–ER junctions. These complexes are assembled within lipid rafts and also contain relevant GPCRs. These complexes must be tethered to the ER in a way that ensures close apposition of ANO1 and IP_3_Rs in order to confer efficient activation of the channel by the Ca^2+^ release. At the same time, such assembly may also protect ANO1 from the ambient Ca^2+^; this is probably why Ca^2+^ influx via the VGCCs is mostly insufficient to activate ANO1 in these neurons. In support of this hypothesis, in several DRG neurons we were unable to activate CaCCs by dialysis of as high as 10 μm free Ca^2+^ through the patch pipette, yet, bradykinin application still activated CaCCs (even in the presence of 10 μm cytosolic free Ca^2+^; X. Jin & N. Gamper, unpublished observations).

Recently in an unbiased SILAC (stable isotope labelling by amino acids in cell culture) proteomics screen of the proteins that physically interact with ANO1 channels in cells, an ER‐localized IP_3_R regulating protein ERLIN1 has been identified as part of the ANO1 interactome (Perez‐Cornejo *et al*. 2012). ERLIN1 (SPFH1) localizes to the ER lipid rafts and physically associates with the IP_3_R in an activity‐dependent fashion to facilitate ER‐associated degradation (ERAD) of the latter (Pearce *et al*. 2007, 2009; Wojcikiewicz *et al*. 2009). The fact that ANO1 interacts with the IP_3_R‐binding protein in the ER further reinforces the idea of ‘special relationships’ of this plasma membrane ion channel with the intracellular Ca^2+^ stores. What remains to be elucidated is whether the interactions between the plasma membrane part of the complex and the ER are static or if they are operated in some way by GPCR triggering or store depletion (e.g. in a way similar to the STIM1–Orai1 complexes assembly in response to store depletion). The latter possibility was recently inferred by Courjaret & Machaca (2014). Our data suggest that ANO1 itself can interact with the IP_3_Rs but we do not know yet if such an interaction is constitutive.

It is worth noting that during evolution the role of anoctamins in plasma membrane–ER interactions may even have preceded their ion channel function. Thus, in yeast an anoctamin orthologue, Ist2, is apparently a tethering protein that holds plasma and ER membranes together to maintain plasma membrane–ER junctions (Stefan *et al*. 2013). In yeast, Ist2 localizes to the ER membrane extending its C‐terminus to the cytosol. A polybasic domain at the very end of the Ist2 C‐terminus acts as an anchor that attaches to the plasma membrane to form a junction with the ER (Stefan *et al*. 2013). ANO1 does not share a high degree of sequence similarity with Ist2 (among the human anoctamins the ANO10 sequence is the most homologous to Ist2) and human ANO1 does not have such a prominent polybasic domain in its C‐terminus as Ist2 does; these features caution against making hasty assumptions. Yet this unforeseen role of yeast anoctamin in supporting the plasma membrane–ER junctions is fascinating in light of what was discussed above.

## Coupling of ANO1 to localized Ca^2+^ sources in smooth muscles

CaCCs are an important excitatory mechanism in vascular smooth muscles. There are a number of Cl^−^ channels expressed in smooth muscle cells (reviewed in Bulley & Jaggar, 2014) and there are at least two types of CaCCs, a ‘classical’ CaCC that displays voltage‐dependent gating and is likely to be mediated by ANO1 (Manoury *et al*. 2010; Thomas‐Gatewood *et al*. 2011) and another Ca^2+^‐dependent Cl^−^ current that requires intracellular cGMP for activation (Matchkov *et al*. 2004, 2005), which possibly depends on bestrophins (Matchkov *et al*. 2008; Bulley & Jaggar, 2014). Several excellent recent reviews discuss the roles and regulation of CaCCs in smooth muscles (Sanders *et al*. 2012; Matchkov *et al*. 2013; Bulley & Jaggar, 2014); therefore, here we will briefly consider only the information concerning the activation of ANO1‐mediated CaCCs by localized Ca^2+^ signalling.

Similar to sensory neurons, smooth muscle cells accumulate relatively high intracellular Cl^−^ conce‐ntrations (∼30–50 mm; Aickin & Vermue, 1983; Owen, 1984; Meyer *et al*. 2002; Bulley & Jaggar, 2014). Accordingly, CaCC activation in smooth muscles induces depolarization and vasoconstriction. The literature on the CaCC activation in smooth muscle cells is rich in examples of multiple Ca^2+^ source coupling mechanisms. Thus, activation of CaCCs by ryanodine receptor (RyR)‐mediated Ca^2+^ release (Bao *et al*. 2008; Zhuge *et al*. 2010), IP_3_R‐mediated Ca^2+^ release (Liu *et al*. 2007), Ca^2+^ influx through VGCCs (Lamb *et al*. 1994), SOCE (Forrest *et al*. 2010; Angermann *et al*. 2012) or other non‐selective Ca^2+^ channels (Bulley *et al*. 2012) have been reported (reviewed in Leblanc *et al*. 2005; Bulley & Jaggar, 2014). However, physiological activation of ANO1‐mediated CaCCs in smooth muscle is likely to depend on co‐localized Ca^2+^ signals since Ca^2+^ sensitivity of ANO1 at physiologically relevant voltages is lower than the physiological range of global [Ca^2+^]_i_ in smooth muscle cells (Knot & Nelson, 1998; Bulley *et al*. 2012). Thus, in cerebral artery smooth muscle cells ANO1 has been found to specifically couple to a Ca^2+^ influx via non‐selective cation channels activated by cell swelling and pressure‐induced membrane stretch but not to VGCCs. The ANO1 activation was blocked by BAPTA but not EGTA, suggesting close association between ANO1 and Ca^2+^ influx channels (Bulley *et al*. 2012).

Another example of close association of ANO1–CaCCs with the Ca^2+^ source in airway smooth muscle cells has been reported by Ronghua Zhuge and colleagues. These authors discovered and characterized in detail a close proximity between CaCCs and RyRs in this type of cells; they further suggested that such association is responsible for the generation of highly specific electrical signals called spontaneous transient inward currents (STICs) that are tightly coupled to RyR‐mediated Ca^2+^ sparks (Bao *et al*. 2008; Zhuge *et al*. 2010). The authors reported that CaCCs were not distributed homogenously in the plasma membrane of the smooth muscle cells but instead were clustered in the proximity of ER‐localized RyRs, possibly in a fashion similar to that reported for nociceptive sensory neurons (Jin *et al*. 2013; see above); the fact that IP_3_Rs and RyRs belong to the same protein family (Amador *et al*. 2013) adds to the analogy. Genetic deletion of ANO1 prevented Ca^2+^ sparks from activating STICs (Zhang *et al*. 2013 *a*). Using a combination of biophysical methods the authors further determined that CaCCs in their smooth muscle preparation were densely clustered in specific spots of the plasma membrane that were less than 600 nm away from the RyR. This proximity established an environment in which CaCCs are exposed to a local [Ca^2+^]_i_ of at least 2.4 μm during the Ca^2+^ spark event. In smooth muscle cells Ca^2+^ sparks activate not only STICs but also spontaneous transient outward currents (STOCs) carried by Ca^2+^‐activated K^+^ channels (Slo, BK; Nelson *et al*. 1995; Bolton & Imaizumi, 1996; ZhuGe *et al*. 1998, 2010; Jaggar *et al*. 2000). It was hypothesized that the physiological role of STICs and STOCs is to stabilize the resting membrane potential of the cell by inducing biphasic membrane potential transients or oscillations that clamp the membrane potential within a negative range and prevent AP generation (Zhuge *et al*. 2010).

Another study has suggested that similar to the DRG neurons, ANO1 is enriched within the caveolin‐1‐containing plasma membrane lipid rafts in the murine portal vein myocytes (Sones *et al*. 2010). Thus, lipid rafts may provide a support for the CaCC–ANO1 clustering observed in STICs studies discussed above. Treatment of these cells with βMCD resulted in membrane re‐distribution of ANO1 and enhanced activation of ANO1‐mediated CaCCs by voltage at relatively high [Ca^2+^]_i_ (0.5 μm). A plausible explanation of this effect is that, similarly to nociceptive DRG neurons, lipid raft‐localized microdomains in smooth muscle cells may play a dual role: (i) providing a mechanism for coupling of ANO1 channels to intracellular stores and (ii) protecting these channels from ‘global’ cytosolic Ca^2+^ elevations. Therefore, lipid raft disruption in smooth muscle cells may have exposed ANO1 channels to global Ca^2+^, making them more easily activated by voltage.

## Activation of ANO‐mediated CaCCs by localized Ca^2+^ signals in other tissues

ANO1‐mediated CaCCs play a robust role in epithelia where these channels (together with the cAMP‐regulated Cl^−^ channel CFTR, and, possibly, other Cl^−^ channels) control secretion. Mechanisms of Cl^−^‐dependent secretion in epithelia are outside the scope of this brief review (for detailed coverage see excellent recent reviews by Jang & Oh, 2014 and Pedemonte & Galietta, 2014). It is worth mentioning though that ANO1‐mediated CaCCs in epithelia are also coupled to G_q/11_–GPCR–PLC–IP_3_ pathways, highlighting once again a functional link between ANO1 and the ER. Thus, the G_q/11_ receptor ligands ATP, substance P, acetylcholine, endothelin 1, angiotensin II and histamine have all been shown to activate CaCCs in epithelial cells of various types by inducing ER Ca^2+^ release (Hartzell *et al*. 2005; Jang & Oh, 2014; Pedemonte & Galietta, 2014).

Another cell type where ANO1 channels play an important role are the interstitial cells of Cajal (ICCs). ICCs are specialized cells of the gastrointestinal tract which control phasic contractions of gastrointestinal smooth muscles by generating waves of electrical activity (‘slow waves’). Release of Ca^2+^ from the IP_3_‐operated ER stores is the fundamental cellular event that initiates pacemaker activity of ICCs (Sanders *et al*. 2006). ICCs are electrically coupled to the smooth muscle cells via gap junctions and, thus, rhythmic depolarizations of ICCs are transferred electrically to smooth muscle cells triggering contractions. It has been suggested that CaCCs are strong contributors to the pacemaker currents in ICCs (Kim *et al*. 2002; Kito *et al*. 2002; Zhu *et al*. 2009). ANO1 is robustly expressed in ICCs (Gomez‐Pinilla *et al*. 2009); moreover, electrical activity and contractility in the gastrointestinal tract have been severely compromised in ANO1 knockout mice (Huang *et al*. 2009; Hwang *et al*. 2009). ANO1 knockout or knock‐down resulted in loss of coordination of both Ca^2+^ transients in ICCs and intestinal contractility (Singh *et al*. 2014). While a precise mechanistic model for the rhythmic activity generated by ICCs remains to be elucidated, it is likely that a close functional coupling between ANO1 channels and the IP_3_Rs will be at the core of any such model.

Interestingly, another anoctamin, ANO2, which also forms CaCCs in a number of cell types, including olfactory neurons (Stephan *et al*. 2009; Rasche *et al*. 2010; Billig *et al*. 2011) and photoreceptors (Stohr *et al*. 2009), apparently has a different Ca^2+^ source preference as compared to ANO1. Huang and colleagues (Huang *et al*. 2012) have shown that in the CA1 region of the hippocampus CaCCs are located postsynaptically in somatodendritic regions of pyramidal neurons; ANO2 has been identified as a likely molecular correlate of these CaCCs. These channels were activated by co‐localized VGCCs and NMDA receptors. Due to the low intracellular Cl^−^ concentration in CNS neurons CaCC activation hyperpolarized the membrane potential; accordingly, in CA1 pyramidal neurons CaCC activation shortened AP duration and raised the threshold, dampened excitatory synaptic potentials and impeded temporal summation. It is not yet clear whether the same CaCCs can co‐localize with both the VGCCs and the NMDA receptors. It is evident, however, that ANO1‐ and ANO2‐mediated CaCCs have different patterns of Ca^2+^ source coupling.

## ANO1 containing signalling complexes: future perspectives

**Figure 3:**
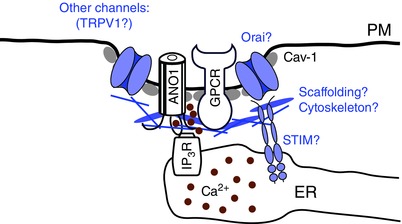
**Hypothetical assembly of ANO1‐containing ER–plasma membrane junctional complexes** Co‐assembly of ANO1, GPCR (i.e. B_2_) and IP_3_ receptors have been supported by experimental data (Jin *et al*. 2013); other hypothetical residents of this signalling complex are shown in blue.

Sensitivities of ANO1 to voltage and [Ca^2+^]_i_ are coupled in such a way that at lower [Ca^2+^]_i_ ANO1 exhibits outwardly rectifying current–voltage relationships whilst at high (several micromolar) [Ca^2+^]_i_ the relationships become linear. In turn, at negative membrane potentials the channel requires much higher [Ca^2+^]_i_ for activation than at positive potentials (Xiao *et al*. 2011; Yu *et al*. 2012). Accordingly, the EC_50_ values for ANO1 activation at negative voltages (e.g. −60 to −100 mV) were reported to be in the range of 3–5 μm while at high, depolarizing voltages the EC_50_ is about tenfold lower (Yang *et al*. 2008; Xiao *et al*. 2011). This means that in cells with negative membrane potentials (such as neurons or muscle cells) ANO1 channels are unlikely to be activated by global Ca^2+^ signals which in most cells do not normally reach micromolar levels. Therefore, in a general sense, physiological activation of ANO1 in excitable cells at rest requires close co‐localization of the channels with the appropriate Ca^2+^ sources. Several examples of such co‐localization have been discussed in this review with one general principle emerging: in many instances ANO1 channels display tight coupling with the Ca^2+^ release channels in the ER. Our data suggest that in sensory neurons ANO1 may physically interact with the IP_3_R (Jin *et al*. 2013), but we do not yet know if these interactions are direct nor if they are constitutive. Likewise, we do not yet know what other components exist within such signalling complexes or what interactions hold these together. Growing evidence suggests that caveolin‐1‐containing lipid rafts may be involved and that these can harbour not only ANO1 but also other components of the signalling complex such as B_2_ receptors and/or PAR‐2 (Jeske *et al*. 2006; Sones *et al*. 2010; Jeske, 2012; Jin *et al*. 2013; Zhang *et al*. 2013 *b*). These complexes are likely to be further supported by scaffolding proteins and cytoskeleton. Indeed, interaction of ANO1 with the ezrin–radixin–moesin network has been identified (Perez‐Cornejo *et al*. 2012). Depending on the cell type, these microdomains may also contain further relevant components, such as Slo channels in smooth muscle cells or TRPV1 channels in sensory neurons. TRPV1 channels are also modulated by B_2_ receptors and PAR‐2 (Chuang *et al*. 2001; Amadesi *et al*. 2004). In addition, both ANO1 (Cho *et al*. 2012) and TRPV1 (Caterina *et al*. 1997) are activated by temperatures within a similar range (42–44 °C), therefore, we can speculate that coupling between Ca^2+^‐permeable TRPV1 and Ca^2+^‐sensitive ANO1 channels may increase the dynamic range of temperature responses in sensory neurons. These microdomains may also include Ca^2+^ release‐activated channel (CRAC) channels as these are needed for refilling the ER Ca^2+^ stores. Moreover, STIM1–Orai1 interactions that bring about CRAC activation (Cahalan, 2009) may provide a further structural link between PM and ER (see Fig. 3 but cf. Courjaret & Machaca, 2014). The list of plausible candidates can surely be continued and we hope that future research will soon identify which of these are genuine. It is also intriguing that the closely related proteins ANO1 and ANO2 apparently have different patterns of coupling to the Ca^2+^ sources. It will therefore be necessary to elucidate the structural background and the physiological significance of such differences.

Finally, it is worth to mention that studying the microdomain localization of proteins has proven to be quite difficult due to: (i) the indirect nature of most available methods for protein–protein interaction (e.g. co‐immunoprecipitation, pull‐down assays etc.); (ii) the resolution limits of most common microscopy techniques (some progress has been made using emerging superresolution methods, but these techniques still suffer from issues (iii) and (iv) below); (iii) antibody specificity issues; (iv) limited suitability of expression systems since overexpressed proteins often ‘disobey’ endogenous localization rules. The latter point is also exemplified by the fact that overexpressed ANO1 channels are usually easily activated by ‘global’ Ca^2+^ elevations. Therefore, a combination of multiple methods is always required for a successful experimental strategy to study localized intracellular signaling, and even then caution is needed in interpreting the results.

## Conclusion

Members of the anoctamin protein family ANO1 and ANO2 mediate CaCC currents in various cell types. These channels have low Ca^2+^ sensitivity and therefore require close association with the source of intracellular Ca^2+^ for reliable activation in their native environment. Growing evidence suggests that ANO1 channels often preferentially couple to the Ca^2+^ release sites in the ER. Examples of such preferential coupling include ANO1‐containing signalling complexes in nociceptive sensory neurons and ANO1‐mediated spontaneous transient inward currents (STICs) in airways smooth muscle cells. In both cases plasma membrane ANO1 channels are found in close proximity to either IP_3_R (nociceptive neurons) or RyRs (smooth muscle cells). Such distinctive coupling may serve to ensure specificity and fidelity of Ca^2+^ signalling pathways in native cells. The mechanisms and physiological significance of specific coupling of ANO channels to the localized Ca^2+^ signals requires further investigation.

## Additional information

### Competing interests

None declared.

### Funding

The work that led up to this review was supported by Wellcome Trust, MRC and National Science Foundation of China.
